# National Dental Association

**Published:** 1900-03

**Authors:** 


### Reports of Society Meetings.

### NATIONAL DENTAL ASSOCIATION.

(Continued from page 123.)

### *Fourth Day.—Morning Session.*

On motion of Dr. J. D. Patterson, the sum of fifty dollars was allowed the committee for the International Dental Congress for incidental expenses, translations, etc. The resignation of Dr. J. B. Monfort was received and accepted.

Dr. Joseph Head, Philadelphia, read a paper entitled "Physiological Reasons for Supposing that Dentine and Enamel in Pulpless Teeth may be Nourished."

In this paper Dr. Head cites the case of a young lady whose slightest systemic derangement was often followed by obstinate pulp-congestion; for the relief of which, after vain trial of other methods for relief, the pulps were successively removed from the left upper first permanent molar and the first and second upper bicuspids on the same side. All of the canals were filled with oxychloride of zinc. Six months later the patient returned suffering from the same trouble in the lower jaw. As the teeth with living pulps failed to respond to tests, the pulpless teeth were also tested, with the result that the teeth of which the canals were filled gave the most intense pain on the application of a hot instrument. The pain being most severe in the molar it was opened, when it was found that the zinc filling had become liquefied, and that the entire dentine had taken on so much inflammation that the patient could not bear to have it touched, the pain being quite as intense as if a live pulp had been distributing nerve-tissue and nourishment.

Sensibility was brought under control by daily applications of chloride of zinc, when they were again filled; but for six months they were still sensitive to heat and cold, but this has since steadily diminished and the patient finally obtained entire relief. The dentine in this case remained sensitive and apparently alive for over a year and a half after the removal of the pulp and filling of root-canals, thus proving that dentine has means of sensitory communication and avenues of nourishment independent of the pulps. Admitting that the dentine is nourished by osmosis through the lacunæ of the cementum, nerve communication between the dentine and the cementum has never been found by the microscope; but the microscope has probably not yet found the half of what it is destined to find; and it is easier to believe that the microscope has overlooked minute sensitory plasma than to believe that sensitory impulses between the dentine and gum are carried on by a Marconi-like system. And since the dentine can be thus nourished, why cannot the enamel be nourished in a similar way? Why should not the phosphate of calcium, or some kindred salt, percolating through the tooth, gradually modify the density of the enamel rods or their sheaths? Excavations show that the enamel of five years is less hard than the enamel of forty years; daily observations show that the enamel grows harder in the course of years, long after the enamel organ has been destroyed. Whether this is due to a further calcification of the rod sheaths or to a change within the enamel molecule cannot be affirmed; but since pulpless dentine has been observed to live and have sensation for over a year, it must have nerve-supply and nourishment independent of the pulp. And if it is possible to the dentine by osmosis through the cementum, it is equally possible to the enamel. When we consider how frequently enamel is known to change its color from permeation by blood-plasma charged with broken-down hemoglobin, not only the possibility, but the probability, of enamel nutrition by osmosis is forced upon our consideration.

Dr. Bryan, of Switzerland, was given the floor, and spoke at some length upon the status of the American dentist in Europe.

At the conclusion of his remarks, Drs. Crouse, Gordon White, and Ottolengui were appointed a committee to work in the direction of securing the removal of the obnoxious requirements in foreign countries.

Dr. D. D. Smith, Philadelphia, next read a paper entitled "The True Status of Pulpless Teeth."

The pulpless tooth sustains unique relations, being a living organ with the greater portion of its substance dead matter. In the minds of the laity it is associated with a blackened crown and rapid decay. By the profession it is usually considered as an erratic organ with sporadic disturbing tendencies impossible to control, and the cause of many aggravated and serious facial neuralgias, dental abscesses, and various active pathological conditions of the mouth and brain.

The utility of a tooth is wholly dependent upon its attachment in the alveolus; a vital living union, partially if not wholly independent of the pulp. The value of a pulpless tooth rests in the fact that there is a source of nutrition maintained independently of pulp action. With the death of the pulp there is death of the whole crown of the tooth. The enamel and dentine are left absolutely without sensitive or nutritive function. Whatever changes take place in the crown subsequent to the death of the pulp are of a disintegrating, retrograde character, but the vitality of the root, except in the dentine, is not interrupted or lessened. The cementum with its membrane is therefore the most important tissue for the conservation of the tooth; its articulation with the alveolus remains unaltered, and the functions of absorption and nutrition are not interfered with. Pulp endowment, in so far as it affects healthful union between root and alveolus, might be dispensed with without detriment. And yet the votaries of certain fashions or fads teach up the union between root and alveolus—extract, mutilate, replant—without regard to the fact that this all-important relation once disturbed is never restored. Such practice is wholly at variance with the laws governing nutrition as exhibited in other parts of the osseous system. Extraction destroys the life of the cementum and the periosteum, depriving the root of cementum and of the only tissue through which restoration to vital union is possible. Extraction and replacement are physiologically incompatible with the office of the tissues involved. Living tissue abhors dead matter and cannot be induced into permanent association, much less vital union. The root portion of an undisturbed pulpless tooth is a vital, living organ, exercising its functions as when the pulp distributed sensation and nutrition to the dentine and enamel. Complete removal of the devitalized pulp-tissue, with proper disinfection of the tubules and intertubular matter, does not interfere with uninterrupted continuation of the normal functions of the tissues which furnish vital attachment between root and alveolus. Such teeth have favorable prognosis in all essential particulars. Pulpless young teeth, those which have lost their pulps before the vascularity of the cementum has been restricted by the contraction of its lacunæ and canaliculi through deposits in its substance, are more subject to cemental accretions than the same teeth with living pulps; but this is not an unfavorable condition, as through it the territory of normal circulation is increased to the benefit of the tooth. A noticeably frequent result of pulp destruction is a closer, firmer union of root and alveolus, due to an increased tendency to deposits of cemental tissue on the surface of the root, and also to the entire obliteration of calcific deposits through pulp influence. A pulpless tooth which has never been the subject of pyorrhœa alveolaris is practically immune to that trouble; in fact, removal of the pulp in the beginning of that disease is attended with the most satisfactory results. With our present methods of crown restoration, the condition of the crown is of little importance. The root should always form the basis of judgment and diagnosis, as upon that depends the permanent comfort and retention of the tooth. The root which has uninjured pericemental connection never questions whether the crown it supports is natural or artificial, but performs all the functions of a tooth with uncomplaining fidelity.

In dealing with a pulpless root devoid of natural crown, there should be free removal of the dentine from the thickest portion of the root, care being exercised not to touch upon the cementum and to leave the typical third practically undisturbed. Pure wood creosote is a specific as a root-dressing, promoting harmonious relations between the devitalized dentine and the living cementum. Crowns having the appearance of the natural organ can be securely applied to any root having the alveolo-dental articulation in a normal physiological condition. For comfortable retention, utility, and desira bility, the prognosis is most favorable.

Dr. Charles L Hungerford, Kansas City, Mo., read a paper entitled "The Physiological Relation of the Dental Pulp to the Economy."

Originally, the dental pulp was a constructive organ, temporarily associated with the economy for the purpose of forming dentine. Its function completed, nature tries to pass it on into other forms, for there is no standing still in nature. Where growth ceases, decay commences, for the involvement of more perfect forms. While a tooth is distinctly a dermal product, it is a differentiation of that derma that gives rise to the three membranes that produce, respectively, enamel, dentine, and cement, each of which grows at the expense of the organ from which it derived its origin, maintaining its existence independently of that organ in exact ratio to that organ's maintaining or losing its original functions. The entire enamel-membrane is used up by the forming enamel; the dentine serves no other purpose than a go-between, supporting the enamel-shield or the outside, connecting it to the more vital cement underneath, but physiologically wholly independent of either. Cut off the crown of the tooth; drill out the dentine to the apex of the root, the cement still lives, and thrives even better than it did before.

There is but one valid argument against pulp removal, and that is the inability to entirely remove it and fill the space. A human tooth does not depend for its physiological relationship with the economy on either the dentine or the enamel, for neither comes in contact with the economy, and, after the removal of the dentine, enamel, and pulp, there is no impairment of the functions of the remaining dental tissues, viz., cement, periosteum, and alveolar process. The dental follicle, having grown enough dentine to give stability, has performed all the physiological function of which it is capable; any further functioning is of an abnormal character, and is followed by disintegration and pain.

On motion, two other papers offered by the Section, "Counter-Irritation," by Dr. W. E. Griswold, and "Recent Advances in Therapeutics," by Dr. A. W. Harlan, were read by title.

In the discussion of the three papers previously read, Dr. M. L. Rhein spoke in advocacy of the views advanced by the essayists as to the advantages obtained by the removal of the pulps of mature teeth when exposed by decay, and depicted the evils resulting from indiscriminate and vain efforts to save the pulps of all teeth. He spoke of the recent valuable histological work of Dr. Fletcher, of Cincinnati, especially his studies of the pericemental alveolar membrane and the importance of the conservation of this portion of the dental organization.

Dr. G. V. Black said that time would not permit of following through all the points of the paper, but it must be borne in mind that we do not yet know all there is to know on any subject. The peridental membrane has not received the close and careful study it demands. The nature of the membrane is not generally understood. Although he has individually given it a great amount of study, he feels that he is not yet familiar with it. There is a set of glands that occupy a position in this membrane that are ignored by the generality of the profession, and the cementum itself is not yet well understood. It is a continually growing body becoming thicker as the tooth is older; it is liable to extraneous growths, becoming thick and nodulous. This may occur at any age, but generally after adult age. The process of detachment and reattachment of the peridental membrane to the tooth is not very generally understood. This is a subject that should be studied, especially by those engaged in moving the teeth, in which process the films of the membrane are severely stretched or entirely detached, either from the alveolar wall or from the cementum, and the space reoccupied by new bone-cells or added cementum.

(*Question.*—As we are so often engaged in moving teeth, will you, as a scientific observer, tell us, does the absorption take place in the cementum or in the bone?)

*Dr. Black.*—The absorption of calcific tissue is from the alveolus principally, though it may be from the cementum also. By the laying down of new cement-tissue, the fibres are unattached, in that the process is the same in replantation. If the conditions are so perfect that this is done on all parts of the root, there will be a perfect reattachment. But when the conditions are not favorable, there will be absorption instead of reattachment, and the tooth will be lost. I agree with the papers that a tooth may very perfectly retain its health and vigor after the removal of the pulp, and its removal is justifiable in many cases. But the question arises, Is a pulpless tooth as good as if the pulp were alive and healthy? In one sense surgery is always an interference with nature. Is the tooth as good? Emphatically NO. But in many cases the only way open is to remove the pulp; but the later in life this is done, the better it is for the tooth. In every case, where a tooth-pulp is destroyed, there is a retrograde change in the dentine, a molecular change; the dentine is not up to its normal strength after the lapse of time. When, from any cause, there is a recession of the pulp, cutting of the dentinal fibrils, the nourishment of that portion of the dentine is cut off; and when you cut into it you will find that it has lost strength, and continues to lose it, even though the pulp remains alive.

I quite agree with the papers as to the complete death of the dentine after the removal of the pulp, but there is an osmosis through the dentine that influences the integrity of the tooth. If the dentinal tubes are open, there is an increase of osmosis from the saliva and we have a retrograde action. If the tubules are not open for the saliva to flow in, then the osmosis is through the cementum to the dentine, which tends towards its integrity. Every time the saliva is allowed to enter a cavity after pulp removal it is injurious to the dentine. Injury is often done by the chemical action of so-called remedies stuffed into cavities. Whenever a case presents in which it is clear the pulp should be devitalized, by no matter what method, nothing should ever be permitted to enter the cavity but what is placed there by design and with a purpose. Seal it up and prevent the entrance of saliva. But do not destroy the pulp in the teeth of children. The pulp is large; the apical foramen is large and open; you will not get the same results that you do in adult teeth. Those who have kept records of cases for many years, and can say just when such a thing was done and how it was done, will tell you that what I tell you is true. If a pulp is removed from a tooth when it is young, it will not stand the wear and tear of a long life.

In closing the discussion of his paper, Dr. Smith maintained the position taken in the paper, that a pulpless tooth was in every sense as good as one with a living pulp; if the root is living and healthy, it makes no difference for functional use whether the crown is a laboratory crown or a natural crown.

Dr. Hungerford said he had nothing to add to what he had said in his paper.

Dr. G. V. I. Brown exhibited and described briefly the illustrations for his paper entitled "Some Cases in Oral Surgery and the Lessons they Teach," but declined to read the paper, the time being too short.

This was the last paper presented. It was announced that the paper which had been accepted by the Sections and presented for reading, but not read, would go to the publication committee. Theories recommended by the Sections, but not presented at this meeting, would be held over for next year by the respective Sections.

After the election, the result of which has been published, and the final routine business, the Association adjourned to meet at Old Point Comfort, Va., June 26, 1900.

Adjourned.

